# Metabolism-related signatures is correlated with poor prognosis and immune infiltration in hepatocellular carcinoma *via* multi-omics analysis and basic experiments

**DOI:** 10.3389/fonc.2023.1130094

**Published:** 2023-02-13

**Authors:** Jiapei Shen, Weijie Sun, Jiaying Liu, Jiali Li, Ying Li, Yufeng Gao

**Affiliations:** Department of Infectious Diseases, The First Affiliated Hospital of Anhui Medical University, Hefei, China

**Keywords:** metabolism, hepatocellular carcinoma, prognostic model, immune infiltration, chemotherapy

## Abstract

**Background:**

Metabolism is an ordered series of biological processes that occur in an organism. Altered cellular metabolism is often closely associated with the development of cancer. The aim of this research was to construct a model by multiple metabolism-related molecules to diagnose and assess the prognosis of patients.

**Method:**

WGCNA analysis was used to screen out differential genes. GO, KEGG are used to explore potential pathways and mechanisms. The lasso regression model was used to filter out the best indicators to construct the model. Single-sample GSEA (ssGSEA) assess immune cells abundance, immune terms in different Metabolism Index (MBI) groups. Human tissues and cells were used to verify the expression of key genes.

**Result:**

WGCNA clustering grouped genes into 5 modules, of which 90 genes from the MEbrown module were selected for subsequent analysis. GO analysis was found that BP mainly has mitotic nuclear division, while KEGG pathway is enriched to Cell cycle, Cellular senescence. Mutation analysis revealed that the frequency of TP53 mutations was much higher in samples from the high MBI group than in the low MBI group. Immunoassay revealed that patients with higher MBI have higher macrophage and Regulatory T cells (Treg) abundance, while NK cells were lowly expressed in the high MBI group. RT-qPCR and immunohistochemistry (IHC) revealed that the hub genes expression is higher in cancer tissues. The expression in hepatocellular carcinoma cells was also much higher than that in normal hepatocytes.

**Conclusion:**

In conclusion, a metabolism-related model was constructed that can be used to estimate the prognosis of hepatocellular carcinoma, and the clinical treatment of different hepatocellular carcinoma patients with medications was guided.

## 1 Introduction

Hepatocellular carcinoma is one of the most commonly diagnosed cancers and is the 2nd major cause of cancer-related deaths (1). According to statistics, Age-standardized incidence and mortality rates (ASRs) of liver cancer worldwide are 9.5 and 8.7 per 100,000 people, respectively. The number of new liver cancer patients will rise by 55% each year in the next 20 years, with 1.4 million people likely to be diagnosed in 2040 (2). Of these, 75-85% of primary liver cancers are Hepatocellular carcinoma (3). Hepatocellular carcinoma (HCC) is mainly caused by chronic HBV or HCV infection, Excessive drinking, rare genetic disorders, and various metabolic diseases (4, 5). Due to the complex etiology of hepatocellular carcinoma, the treatment methods of hepatocellular carcinoma have great differences (6). Therefore, new biomarkers and prognostic models are needed for the precise management of individuals.

Metabolism is an orderly sequence of biological changes that take place in an organism to sustain life (7, 8). Several studies have shown that alterations in cellular metabolism are often closely associated with the development of cancer (9, 10). One study found hepatocellular carcinoma was promoted with fat by inducing glucose metabolism in unconverted hepatocytes (11). And HDAC11 can regulate the glycolytic process through LKB1/AMPK signaling pathway, which can maintain hepatocyte cancer stemness (12). In addition, PRMT6-ERK-PKM2 plays an important role in tumorigenesis. It also provides an important connection between tumor and glucose metabolism (13).. However, current studies on liver cancer and metabolism are often limited to a single molecule, while studies on multiple metabolism-related genes and liver cancer are still scarce.

The aim of this study is to integrate multiple metabolism-related key genes to construct an excellent model to diagnose and assess patient prognosis. The correlation of the model with immunotherapy, drug sensitivity allows to give different treatment regimens for different populations. In addition, we validated these results by multi-omics analysis and basic experiments.

## 2 Materials and methods

### 2.1 Data source

The Cancer Genome Atlas (TCGA) (https://portal.gdc.cancer.gov/) program was launched by the National Cancer Institute (NCI) and the National Human Genome Research Institute (NHGRI), and currently studies 36 cancer types in total (14). 374 LIHC samples and 50 normal samples were included for study, with clinical data including gender, age, stage, grade, survival time, survival status, etc. In addition, the corresponding mutation data was downloaded of hepatocellular carcinoma for subsequent mutation analysis. The ICGC database (https://dcc.icgc.org/projects/LIRI-JP) is an international tumor genome collaborative group, a global collaborative database containing samples from different countries and regions. We downloaded data from it for liver cancer as a validation cohort. The GEO dataset (GSE45267) was used for external validation (https://www.ncbi.nlm.nih.gov/).

### 2.2 Weighted gene co-expression network analysis

WGCNA is to explore whether there is co-expression between genes and to divide a certain cluster of co-expressed genes into a module based on certain values, so that different clusters of genes clustered together are divided into different modules. Metabolism-related genes were used to construct the weighted correlation network, where modules that differed between cancer and paracancer were selected for further analysis (15).

### 2.3 Functional enrichment analysis

KEGG is a database for studying biological functions from genomic and molecular level information. Its PATHWAY sub-database integrates current knowledge in molecular interaction networks and allows prediction of pathways enriched by differential genes. The Gene Ontology (GO) database divides the functions of genes into three components: cellular component (CC), molecular function (MF), and biological process (BP). To explore the underlying biological processes and signaling pathways associated with the acquisition of differential genes.

### 2.4 Model construction

A risk score model was constructed based on the integrated role of key genes in liver cancer to comprehensively evaluate the function of these molecules in patient prognosis. The model was construct by lasso-cox regression analysis. The metabolic index (MBI) for each patient with HCC was computed with the equation:

MBI = ∑ (Expression level of Gene i × coefficient i)

The TCGA dataset is the training set and the ICGC is the validation set. Kaplan-Meier curves were used to investigate survival situation between different MBI groups of patients with liver cancer in the training and validation set. Risk-survival curves were used to explore the survival and mortality of the samples in different MBI groups, and the differences in the key genes that were modeled between the two groups. The roc curve is used to determine the effectiveness of this prediction model.

### 2.5 Immunoassay and drug sensitivity analysis

The abundance scores of 16 immune cells and 13 immune-associated terms in each sample were assessed with ssGSEA method. The estimate algorithm was used to calculate the immunescore, stromalscore, and Estimatescore in the tumor microenvironment. The IMvigor210 dataset was used to assess the efficacy of immunotherapy. We used the “oncopredict” package to differentiate the sensitivity of the different groups to the drug, of which a total of 198 chemotherapeutic agents could be evaluated. We screened the most sensitive drugs from them (16).

### 2.6 Immunohistochemistry

Human Protein Atlas (HPA) (https://www.proteinatlas.org/) is a freely available public database (17). This database investigates the expression of proteins at the protein level in various human tissues and organs. It contains the IHC profile in normal and tumor tissues. The expression of key genes in normal and tumor tissues was investigated.

### 2.7 Sample collection

Ten pairs of HCC tissues and normal tissues were provided by the First Affiliated Hospital of Anhui Medical University, and the samples were stored at -80°C for a long time. The ethics committee of the First Affiliated Hospital of Anhui Medical University approved the study.

### 2.8 Real-time quantitative PCR

The specific experimental procedure is described previously (18). TRIZOL (#15596026, Invitrogen, USA) was used to extracted RNA according to manufacturer’s instructions. According to protocol, cDNA was derived from RNA by reverse transcription by PrimeScript™ RT Master Mix (Takara Bio, Japan). TB Green^®^ Premix Ex Taq™ II (Tli RNaseH Plus), Bulk (#RR820L, Takara, Japan) was used to perform RT-qPCR according to the manufacturer’s instructions. RT-qPCR reaction program: Preheat: 95°, 30; Denaturation: 95°, 30s; Protein refolding: 55°, 30s; Cycle: 72°, 60s, 40 times; Termination: 72°, 5min. The primer sequences are as follows: ATIC-F: ACCTGACCGCTCTTGGTTTG, ATIC-R: TACGAGCTAGGATTCCAGCAT; G6PD-F: CGAGGCCGTCACCAAGAAC, G6PD-R: GTAGTGGTCGATGCGGTAGA; GAPDH-F: GGAGCGAGATCCCTCCAAAAT, GAPDH-R: GGCTGTTGTCATACTTCTCATGG.

### 2.9 Data statistics

Analysis of differences was performed using the Wilcoxon test. The correlation analysis was based on the Spearman correlation test. Kaplan-Meier analysis was used for survival analysis. R package “survival” was used to perform Cox regression analysis, along with hazard ratio (HR) and 95% confidence interval (CI). P < 0.05 was regarded as statistically significant. R software (version 4.1.2) performs statistical analysis and plotting.

## 3 Result

### 3.1 WGCNA screening for differential genes

**Figure 1:**
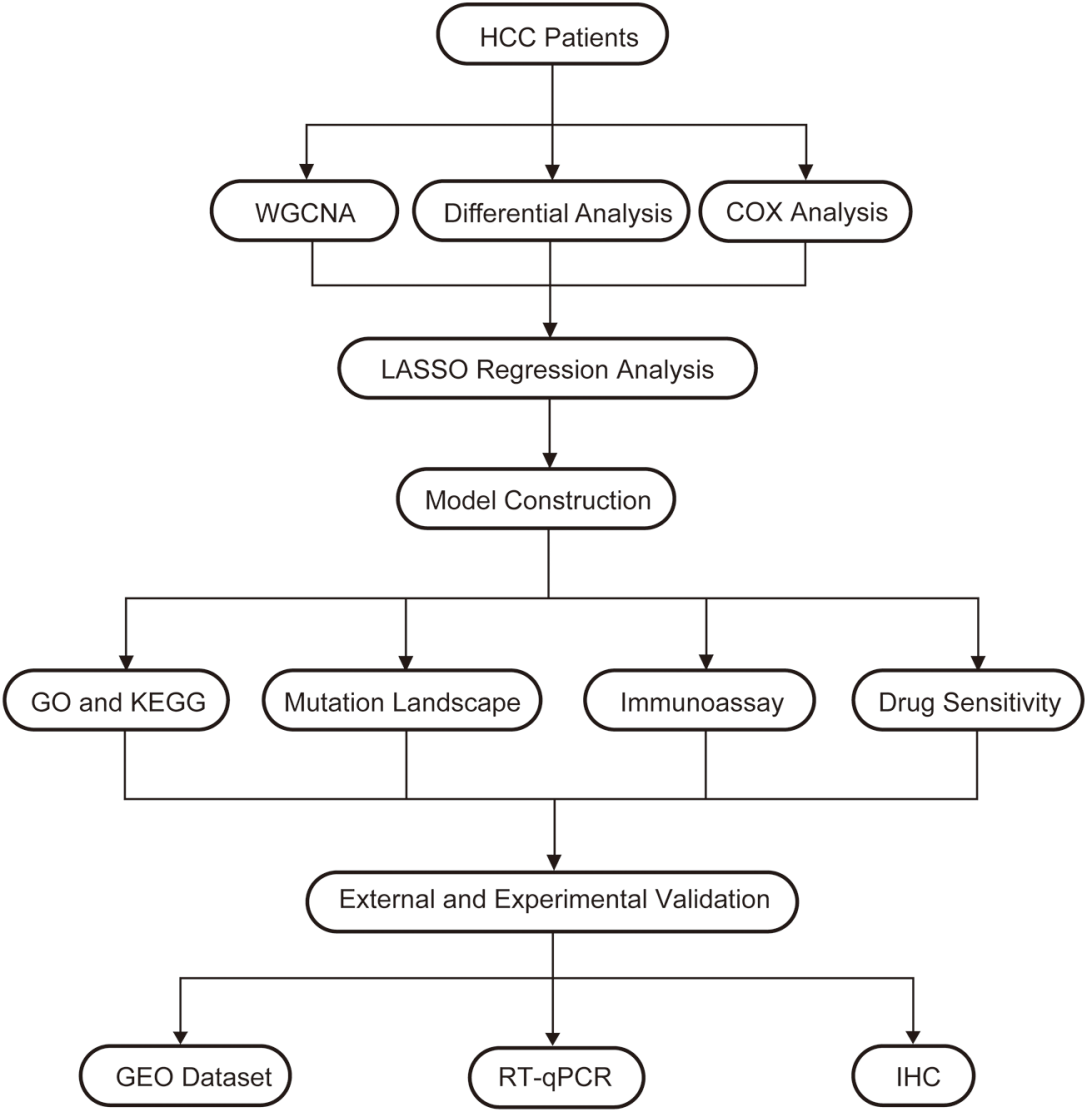
A flow chart of the manuscript.

**Figure 2:**
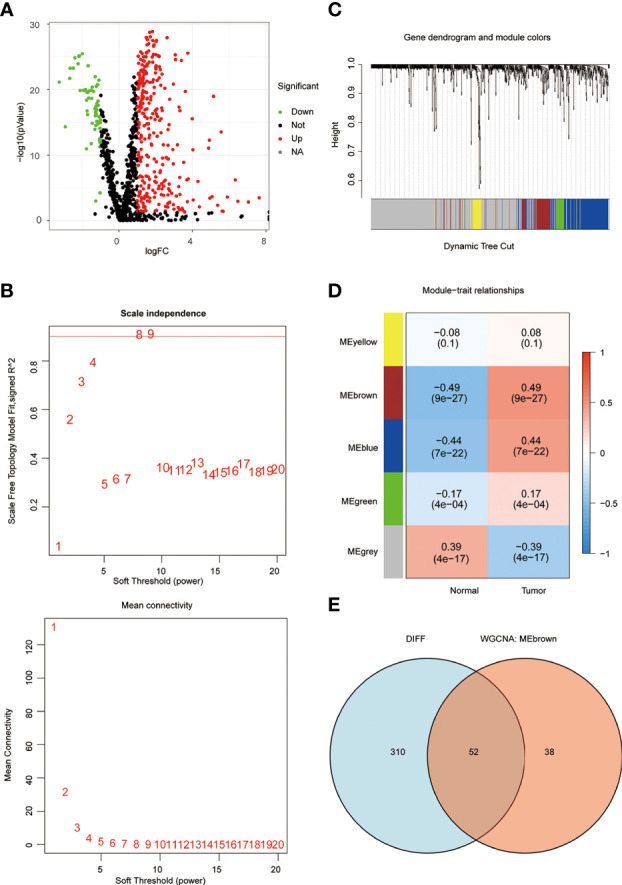
WGCNA screening for key genes. **(A)** The volcano plot clearly shows the differential genes, with a total of 362 differential genes. **(B)** the optimal soft threshold 8 is used to construct the co-expression network. **(C, D)** The genes were divided into 5 modules by co-expression network, and the P-value and correlation coefficient of each module were clearly marked. **(E)** VENN plot showing the intersection of differential genes and the MEbrown module of WGCNA with 52 genes.

In order to understand the framework and logic of the article, a flowchart was drawn (**Figure 1**). 947 metabolism-related genes were collected from the GSEA database, and a total of 362 genes were differential genes after performing differential analysis, as can be clearly seen in the volcano plot (P < 0.05, |log 2 FC| > 1) (**Figure 2A**). After calculation, the optimal soft threshold 8 is used to construct the co-expression network (**Figure 2B**). After dividing the genes into different modules, it is used to draw the gene clustering tree (**Figure 2C**). The clustering was performed by WGCNA, and we can see that the genes were divided into 5 modules, among which the MEbrown module was selected for subsequent analysis, with a total of 90 genes (**Figure 2D**). After intersecting the differential analysis and the genes selected by WGCNA, 52 genes were used for our subsequent analysis (**Figure 2E**).

### 3.2 Construction of lasso regression model

**Figure 3:**
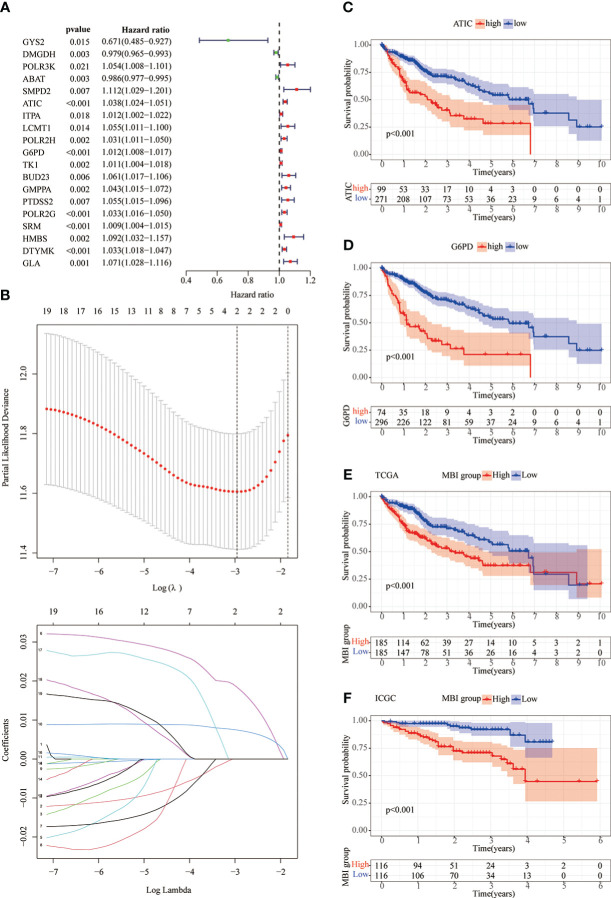
Lasso regression model and KM prognostic analysis. **(A)** Univariate COX prognostic analysis revealed genes with prognostic value. **(B)** The LASSO regression model calculated the best lamda value of 2 and selected the best modeled genes. **(C, D)** KM analysis shows poorer prognosis when both ATIC and G6PD are genes with high expression. **(E, F)** In the TCGA and ICGC databases, KM analysis identified a poorer prognosis in the high MBI group.

As previously described, we performed a univariate COX prognostic analysis of these differential genes, 19 of which were statistically significant (P < 0.05) (**Figure 3A**). Next, a further LASSO regression model calculated the optimal lamda value of 2, where ATIC and G6PD were used to construct the model (**Figure 3B**). KM analysis was used to analyze the prognosis of the two genes, and it can be seen from the graph that patients in the high gene expression group had a poorer prognosis (**Figures 3C, D**). We used the TCGA database and applied the model to evaluate every patient’s MBI. Patients were grouped equally to two teams according to MBI, and it was found that high MBI patients had a worse prognosis (**Figure 3E**). The model was further validated using the ICGC dataset and the outcome is in agreement with the above (**Figure 3F**). Further risk curves were then plotted, and both the TCGA database and the ICGC database found higher mortality rates for patients at higher MBI (**Supplementary Figure 1A-D**).

**Figure 4:**
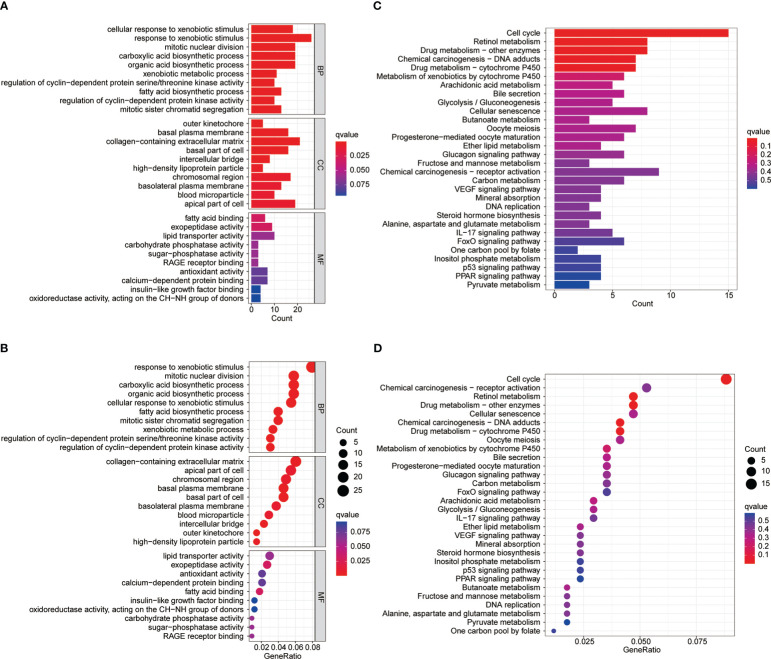
GO and KEGG enrichment analysis. **(A-B)** Gene Ontology functional enrichment analyses for differentially expressed genes. **(C-D)** KEGG pathway enrichment analyses for differentially expressed genes.

To explore the differences between differential MBI groups, GO and KEGG analysis were performed using differential genes. Among them, BP was mainly enriched to mitotic nuclear division, CC enrichment to chromosomal regions and collagen-containing extracellular matrix, and MF is mainly enriched to lipid transporter activity (**Figures 4A, B**). KEGG analysis revealed that the enriched pathways were mainly cell cycle, chemical carcinogenesis and cellular senescence (**Figures 4C, D**).

### 3.3 Prognostic value of MBI

**Figure 5:**
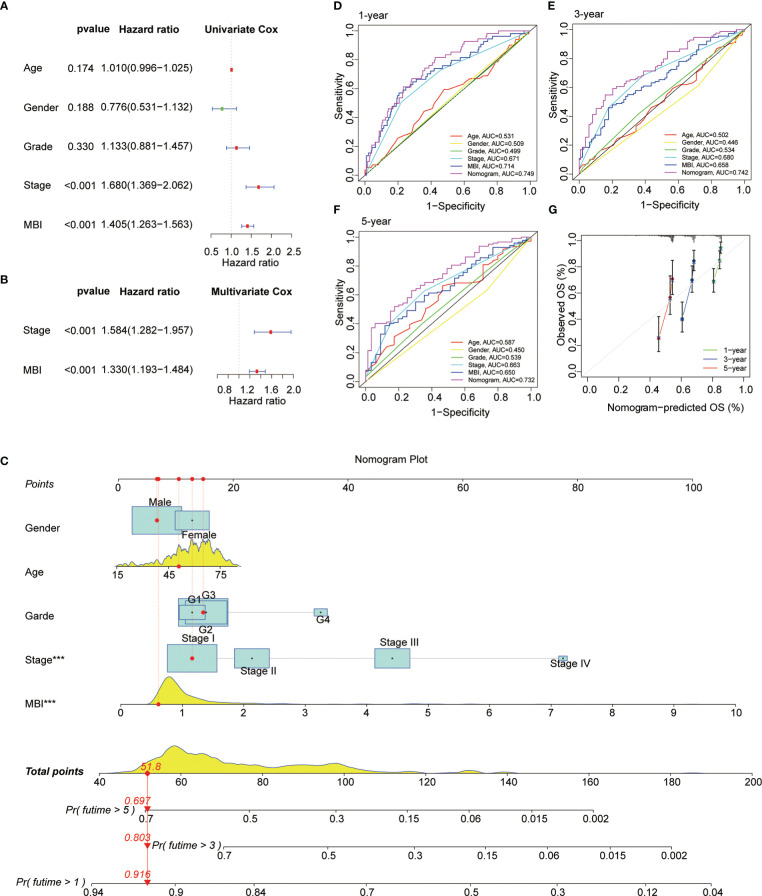
Prognostic Value and Clinical Value of MBI. **(A)** Univariate COX analysis identified MBI as a risk factor. **(B)** multivariate COX analysis identified MBI as a risk factor. **(C)** The nomogram constructed to predict the probability of patient mortality. **(D–F)** ROC curves show that both MBI and nomogram scores are good predictors. **(G)** The calibration curve results found that the model works well. ***P < 0.001.

Subsequently, we performed a COX prognostic analysis of the clinical characteristics and MBI of these patients. Among them, staging and MBI were statistically significant in both univariate and multivariate analyses (**Figures 5A, B**). Considering the clinical use of the model, we further plotted the nomogram. MBI were included in addition to the clinical information of the patients (**Figure 5C**). The ROC curves analyzed the efficacy of the model at 1, 3 and 5 years and found that all were well evaluated. Considering that the inclusion of clinical information may be more effective for the model, we calculated the Nomogram score. and the ROC curve was found to be better when it was evaluated (**Figures 5D, F**). The calibration curve results found the model to be good (**Figure 5G**).

### 3.4 Mutation landscape of different MBI groups

**Figure 6:**
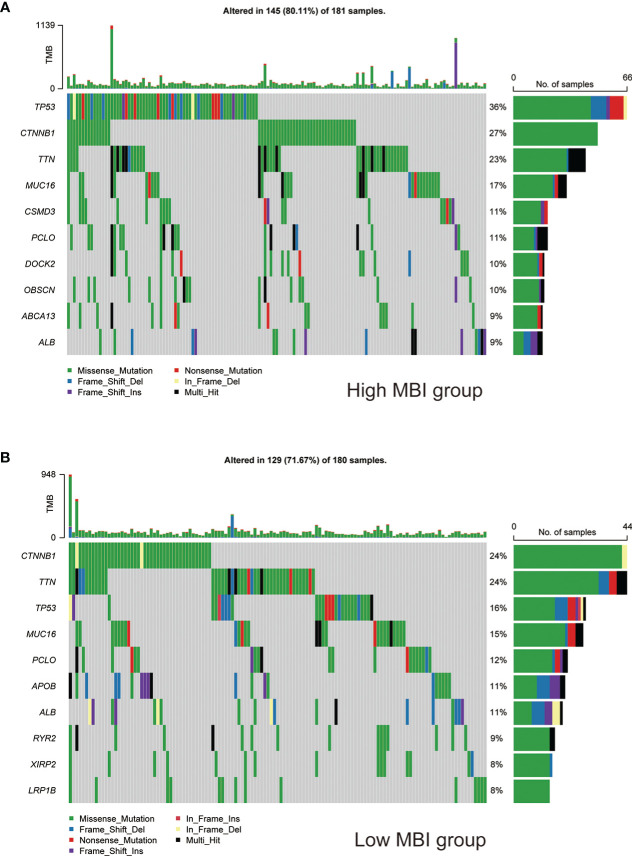
Mutation landscape of different MBI groups. **(A)** Mutation types, mutated genes and mutation frequencies in patients in the high MBI group. **(B)** Mutation types, mutated genes and mutation frequencies in patients in the low MBI group.

To study the profile of mutations, we mapped the major mutated genes and frequencies in the different MBI groups. The predominant mutation type in both MBI groups was Missense Mutation. And TP53, CTNNB1, and MUC16 were mutated more frequently in high MBI patients. Among them, the TP53 mutation frequency was 36% in the high MBI group, while it was 16% in the low MBI group (**Figures 6A, B**).

### 3.5 Immunoassay and drug sensitivity analysis

**Figure 7:**
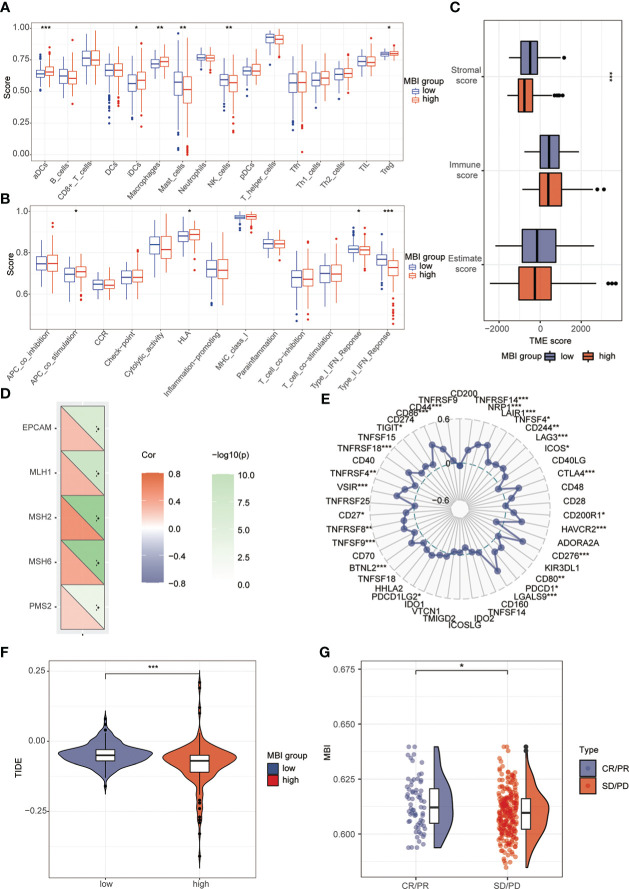
Analysis of immune cells, immune terminology and immunotherapy. **(A)** Immunoassay revealed the expression of aDCs, iDCs, Macrophages, Treg in the high MBI group. Mast_cells and NK_cell are mainly expressed in the low MBI group. **(B)** APC_co_stimulation and HLA are mainly expressed in the high MBI group. While Type_Ⅰ_IFN_Response and Type_Ⅱ_IFN_Response are mainly expressed in the low MBI group. **(C)** Lower Stromalscore in the high MBI group in the tumor microenvironment. **(D)** MMR is correlated with MBI, with MSH2 having the highest correlation with MBI. **(E)** Immune checkpoint analysis revealed a significant correlation between MBI and PDL1, PD1, and CTLA4 etc. **(F)** The TIDE score is lower in high MBI patients. **(G)** Patients with better immunotherapy outcomes had higher MBI in IMvigor210 dataset. ***P < 0.001, **P < 0.01, *P < 0.05.

**Figure 8:**
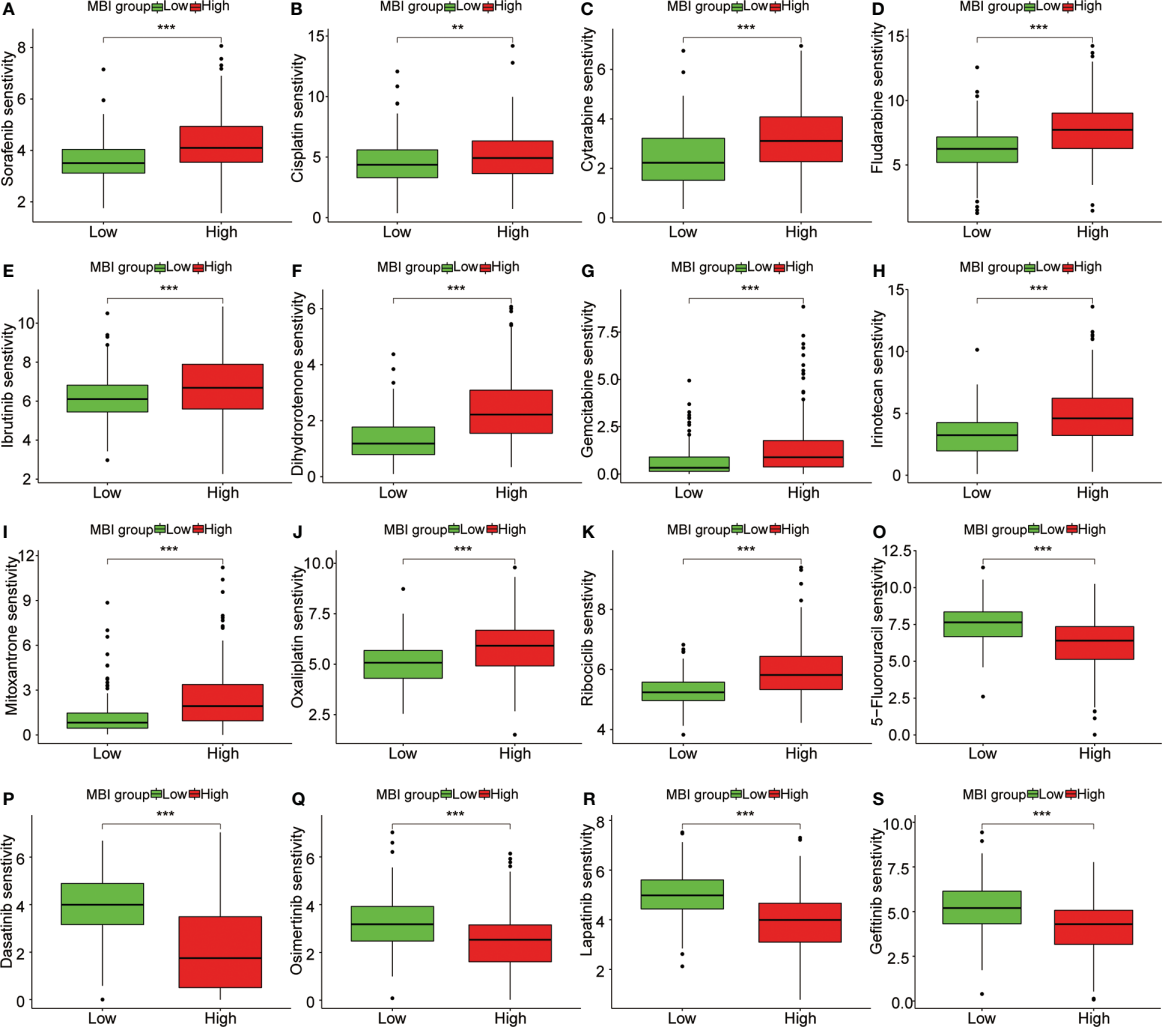
Sensitivity analysis of multiple chemotherapeutic agents. **(A-K)** Sorafenib, Cisplatin, Cytarabine and Fludarabine, Ibrutinib, Dihydrorotenone, Gemcitabine, Irinotecan, Mitoxantrone, Oxaliplatin and Ribociclib had higher IC50 values in the high MBI group. **(O-S)** 5-Fluorouracil, Dasatinib, Osimertinib, Lapatinib, and Gefitinib had higher IC50 values in the low MBI group. ***P < 0.001, **P < 0.01.

To explore the relationship between MBI and immune cells, we investigated the expression of 16 immune cells. Multiple immune cells differed significantly between high and low MBI groups. Among them, aDCs, iDCs, Macrophages, and Treg have higher expression in high MBI patients. And Mast cells, NK cell were mainly expressed in the low MBI group (**Figure 7A**). Subsequently, we further evaluated the differences in immune-related functions in high and low MBI groups, and we found that APC_co_stimulation and HLA were predominantly expressed in the high MBI group. While Type_I_IFN_Response and Type_II_IFN_Response was expressed in low MBI patients (**Figure 7B**). Next, we found a lower Stromalscore in the high MBI group in the tumor microenvironment (**Figure 7C**). Considering the relationship between MBI and immunotherapy, we evaluated the relationship between MBI and MMR. MMR all correlated with MBI, with MSH2 having the highest correlation with MBI (**Figure 7D**). Interestingly, immune checkpoint analysis revealed a significant correlation between MBI and PDL1, PD1, and CTLA4 (**Figure 7E**). The model was found to be a better predictor of the effect of immunotherapy through the dataset, where the TIDE score was lower in high MBI patients (**Figure 7F**). Afterwards, we found that patients with better immunotherapy outcomes had higher MBI (**Figure 7G**). Subsequently, we investigated the sensitivity of different chemotherapeutic agents in different MBI groups, and we found IC50 values of Sorafenib, Cisplatin, Cytarabine, Fludarabine, Ibrutinib, Dihydrorotenone, Gemcitabine, Irinotecan, Mitoxantrone, Oxaliplatin and Ribociclib were higher in the high MBI group. While 5-Fluorouracil, Dasatinib, Osimertinib, Lapatinib, and Gefitinib had higher IC50 values in the low MBI group (**Figures 8A–S**).

### 3.6 External and experimental validation of key gene expression

**Figure 9:**
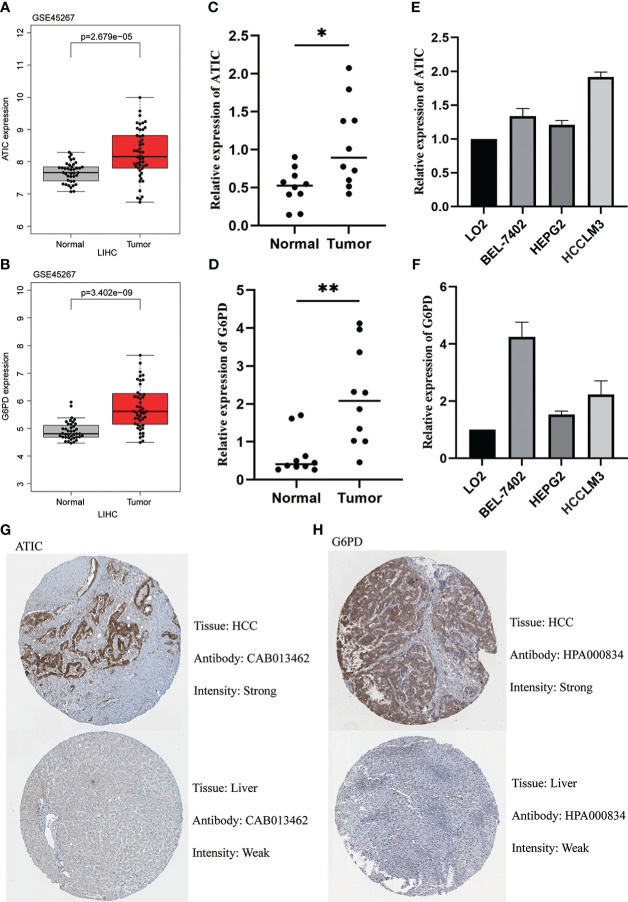
Validation of ATIC and G6PD expression in Tissues and Cell Lines. **(A, B)** GEO database (GSE45267) demonstrates that ATIC and G6PD are significantly more expressed in tumor tissues than in normal tissues. **(C, D)** qPCR demonstrates that ATIC and G6PD are significantly more expressed in tumor tissues than in normal tissues. **(E, F)** qPCR demonstrated that the expression of ATIC and G6PD was significantly higher in hepatocellular carcinoma cells than in normal hepatocytes. **(G, H)** IHC demonstrated that the expression of ATIC and G6PD was significantly higher in tumor tissues than in normal tissues.

We first found that both ATIC and G6PD were significantly higher expression in cancerous tissue through the GEO database (**Figures 9A, B**). The subsequent qPCR performed on 10 pairs of hepatocellular carcinoma and paraneoplastic tissues used both revealed higher expression of these two key genes in the tumor tissues (**Figures 9C, D**). Immediately after, we further performed qPCR validation using LO2 normal hepatocytes and three types of hepatocellular carcinoma cells, BEL-7402, HEPG2 and HCCLM3, and we found hub genes were higher in cancer cells than in normal hepatocytes (**Figures 9E, F**). Finally, we observed the immunohistochemistry of these two critical genes. The results were basically consistent with the above, that is, higher expression in HCC tissues (**Figure 9G, H**).

## 4 Discussion

The treatment of hepatocellular carcinoma is still mainly surgical, but the recurrence rate of tumors in patients with intermediate and advanced stages is extremely high (19). With the advent of the era of tumor immunotherapy, immune checkpoint inhibitors have been used as one of the new effective approaches for tumor treatment (20). Therefore, it is difficult to know whether immunotherapy is effective for a particular patient, and how to screen this group of responding patients is a clinical problem that needs to be solved. We used metabolism-related genes to construct the model and also studied the correlation between the model and immunity to screen for this population of patients.

In this study, AITC and G6PD were screened as key genes for the model by WGCNA as well as lasso-cox based on TCGA transcriptome data and clinical information. It was shown that G6PD is activated by TSP50 along with the development of hepatocellular carcinoma (21). In addition, Wang and Zhao et al. found that G6PD could be used for building models of hepatocellular carcinoma (22, 23). All of the above studies indicate that G6PD and the development of hepatocellular carcinoma are closely related. The bifunctional enzyme ATIC can promote the proliferation of liver cancer (24). Moreover, ATIC can also be used as a prognostic biomarker for hepatocellular carcinoma (25, 26). These studies further confirmed that these two genes are good indicators for the construction of prognostic models for liver cancer.

We further performed functional enrichment analysis. Where BP is enriched to mitotic nuclear division, it has been found that mitotic transmission disruption usually leads to aneuploid offspring production. Aneuploidy is a general characteristic of tumor cells (27, 28). Interestingly, KEGG is enriched to Cell cycle, Chemical carcinogenesis, Cellular senescence and other pathways. Matthews’ study found that impairing cell exit from the cell cycle to allow continuous cell division can lead to cancer development (29–31). Cellular senescence is a condition in which the cell cycle is stalled. The senescent state leads to the development of cancer by keeping the cells alive (32, 33). These studies further confirm our results and show that MBI is a good predictor. Further exploration of the correlation of MBI and prognosis, we performed univariate and multivariate cox analyses and found MBI to be a risk factor. In different years, the ROC curve showed that the prediction efficiency of the model was better. Further nomograms can help to score clinical patients so that different treatment plans can be given to patients with different scores. Subsequently, to explore the differential profile in different MBI groups, we analyzed the mutation types and mutation frequencies. The TP53 mutation frequency was much higher in samples of the high MBI patients. It was shown that Survival of liver cancer cells increases with increasing mitochondrial fission wtih coordinated regulation of ROS-regulated NF-κB and TP53 pathways (34). And TP53 are significantly mutated in liver cancer (35, 36). Several studies above have demonstrated that mutations in are strongly correlated with hepatocellular carcinoma from multiple clinical and basic perspectives. So, the high MBI group has a poorer prognosis for this reason.

To explore the relevance of MBI and immunity. We studied 16 immune cells and 13 immune-related terms. Previous studies have demonstrated that Macrophages polarize under the influence of tumor microenvironment and form tumor-associated macrophages (TAM). And the abundance of TAM in tumors is closely associated with poor prognosis (37). Our study revealed that macrophage abundance was higher in the high MBI patients. This also explains that high MBI patients had a worse prognosis. Togashi’s study showed that Treg cells expressing FOXP3 suppress aberrant immune responses against autoantigens and also suppress anti-tumor immune responses. Large numbers of Treg cells infiltrating into the tumor tissue usually have a poor prognosis (38). This is broadly in line with our results that the higher MBI patients had higher Treg. In addition, we found that NK cells were lowly expressed in patients in the high MBI group. According to others, if cell surface markers are correlated with carcinogenic transformation, NK cells can quickly eliminate them. This property is unique among immune cells, and their ability to enhance antibody and T cell responses supports the role of NK cells as anti-cancer agents (39). This further confirms our results. After that, we observed that the interferon response was mainly expressed in the group with low MBI. Interestingly, Boukhaled et al. found that IFN contributes to antitumor immune quality and immunotherapeutic response (40, 41). Subsequently, we predicted the effect of immunotherapy by analyzing the TIDE scores of the high and low MBI groups. In addition, we found that MBI was closely associated with immunotherapy by MMR and immune checkpoint analysis. In particular, the correlation between CTLA4 and MBI was extremely high, which indicates that MBI is of great value in guiding clinical immunotherapy. Our validation using the dataset revealed that patients with high MBI had better outcomes after immunotherapy. Our study also found significant differences between the high and low MBI groups across multiple chemotherapeutic agents. These results can further guide our clinical use of medications. Finally, we verified the expression of key genes by qPCR in tumor and normal tissues and in hepatocytes and hepatoma cells. This was confirmed by further IHC results.

This study has important clinical applications. Our metabolism-related genes screened by WGCNA combined with differential analysis and prognostic analysis were more credible. Exploration of GO and KEGG helps us to understand the reasons for the difference between high and low risk groups. It helps to point the way for our next study. Moreover, MBI calculated by the model constructed by lasso-cox is a credible and independent biological marker to predict the prognosis of patients of hepatocellular carcinoma. Studying the correlation between MBI and immune and drug name susceptibility can be a useful indicator to assess the efficacy of immunotherapy and chemotherapy in patients.

There are several limitations to our study that need to be acknowledged. First, this study is an analysis using a public database and lacks validation of our own cohort. We will further investigate these hub genes in our own cohort of hepatocellular carcinoma data. Secondly, the downstream target genes of these two genes were not explored further, which also needs our further study afterwards.

## 5 Conclusion

In brief, we constructed a metabolism-related model. We hope that this model can be used as a reference for predicting patient survival and guiding related treatments for patients with liver cancer.

## Data availability statement

The datasets presented in this study can be found in online repositories. The names of the repository/repositories and accession number(s) can be found in the article/**Supplementary Material**.

## Ethics statement

The studies involving human participants were reviewed and approved by The ethics committee of the First Affiliated Hospital of Anhui Medical University. The patients/participants provided their written informed consent to participate in this study.

## Author contributions

YG have constructed and devised the research. JS and WS performed data analysis and wrote the manuscript. JYL and YL analyzed the data. JLL acquired samples and performed the experiments. All authors contributed to the article and approved the submitted version.

## Supplementary material

Supplementary Figure 1 Risk curves for different data sets. **(A, B)** TCGA database analysis finds higher mortality in patients in high MBI group. **(C, D)** ICGC database analysis finds higher mortality in patients in high MBI group.
