# Supplementary figures and images for: Metabolism-related signatures is correlated with poor prognosis and immune infiltration in hepatocellular carcinoma via multi-omics analysis and basic experiments
Source: Front Oncol. 2023 Feb 13;13:1130094. doi: 10.3389/fonc.2023.1130094 (PMC9969091; doi:10.3389/fonc.2023.1130094)

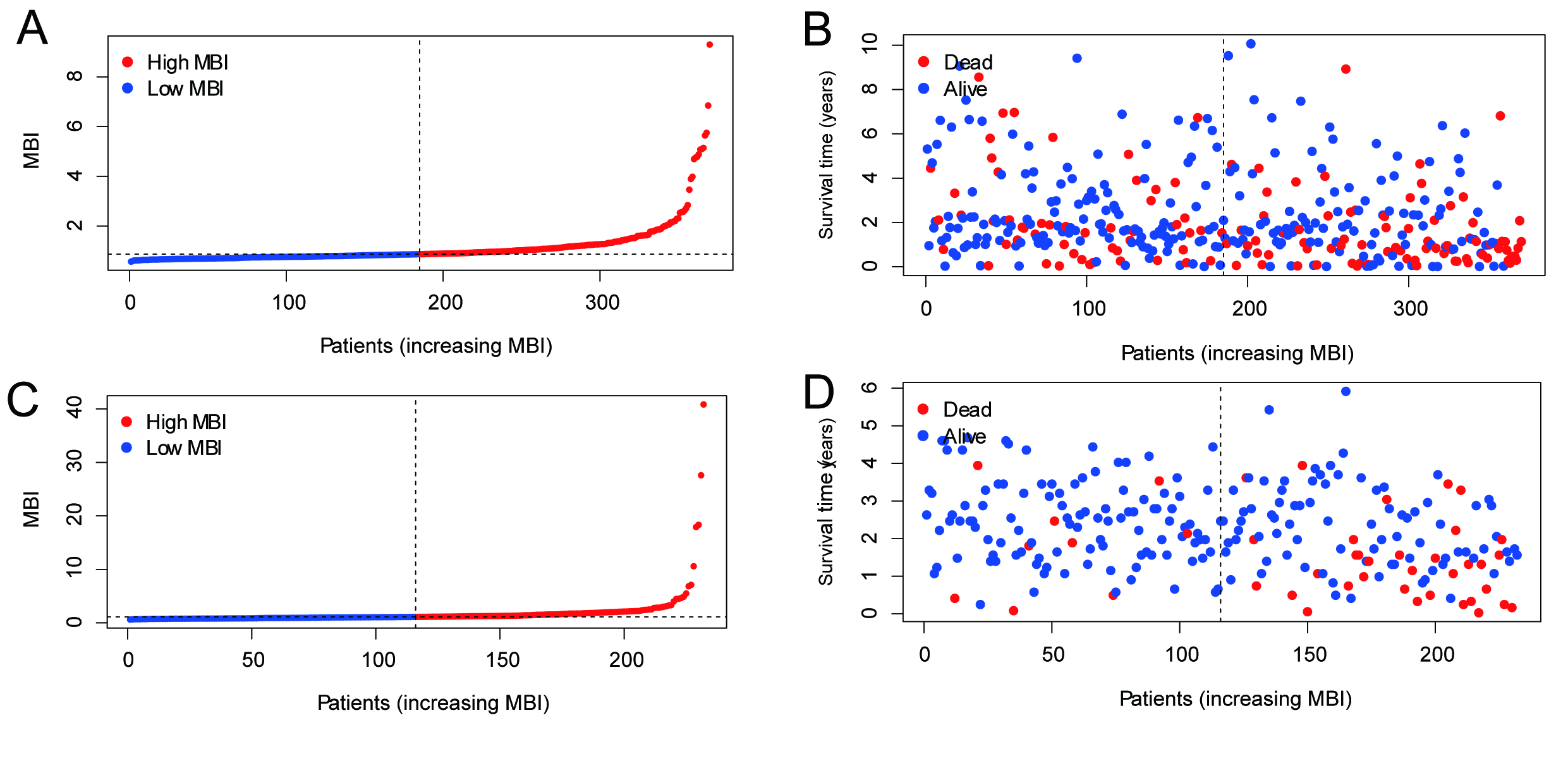

Supplement: Supplementary Figure 1 — Risk curves for different data sets. (A, B) TCGA database analysis finds higher mortality in patients in high MBI group. (C, D) ICGC database analysis finds higher mortality in patients in high MBI group. [file Image_1.tif]
